# COVID-19 Monitoring and Response Among U.S. Air Force Basic Military Trainees — Texas, March–April 2020

**DOI:** 10.15585/mmwr.mm6922e2

**Published:** 2020-06-05

**Authors:** Joseph E. Marcus, Dianne N. Frankel, Mary T. Pawlak, Theresa M. Casey, Rebecca S. Blackwell, Francis V. Tran, Mathew J. Dolan, Heather C. Yun

**Affiliations:** ^1^Infectious Disease Service, Brooke Army Medical Center, Joint Base San Antonio, Texas; ^2^Trainee Health Surveillance, 559 THLS, Joint Base San Antonio-Lackland, Texas; ^3^Trainee Health, 559 THLS, Joint Base San Antonio-Lackland, Texas.

The coronavirus disease 2019 (COVID-19) pandemic has resulted in substantial morbidity and mortality since it was first described in December 2019 (*1*). Based on epidemiologic data showing spread in congregate settings (*2*–*4*), national, state, and local governments instituted significant restrictions on large gatherings to prevent transmission of disease in early March 2020. This and other nonpharmaceutical interventions (NPIs) have shown initial success in slowing the pandemic across the country (*5*). This report examines the first 7 weeks (March 1–April 18) of implementation of NPIs in Basic Military Training (BMT) at a U.S. Air Force base. In a population of 10,579 trainees, COVID-19 incidence was limited to five cases (47 per 100,000 persons), three of which were in persons who were contacts of the first patient. Transmission of symptomatic COVID-19 was successfully limited using strategies of quarantine, social distancing, early screening of trainees, rapid isolation of persons with suspected cases, and monitored reentry into training for trainees with positive test results after resolution of symptoms.

BMT is the first step in the accession of airmen into the USAF. Approximately 40,000 new airmen are trained each year at Joint Base San Antonio-Lackland (JBSA) in Texas with an average of approximately 800 trainees arriving per week. Approximately 75% of incoming trainees are male, and most are in their late teens or early 20s. These trainees are prescreened for underlying medical conditions and are generally in good overall health. Training involves classroom lectures, small group activities, and field exercises. Each training cohort (flight) consists of 50 persons who live in communal, open-bay quarters and perform all daily and training activities as a group. For accountability and safety purposes, trainees are never alone, performing every activity with at least one fellow trainee. In recent decades, outbreaks of respiratory illnesses caused by pathogens such as adenovirus serotype B14 in 2007 have occurred during BMT, resulting in head-to-toe bunk arrangements, regular cleaning of shared equipment, and active syndromic surveillance for respiratory illness (*6*).

## Diagnostic Testing Strategy

The initial testing approach for COVID-19 at the Air Force base was based on CDC guidelines (*7*). Initially, trainees who reported as ill to the medical officer on duty (sick call) were evaluated. Trainees were eligible for testing for SARS-CoV-2, the virus that causes COVID-19, only if they reported both symptoms (including cough, fever, or shortness of breath) and either exposure to a person known to have COVID-19 or travel from a high-transmission area. Using these criteria, from March 1 to March 15, two patients were tested for COVID-19. On March 16, the testing criteria became entirely symptom-based with no exposure prerequisite. All trainees underwent an entry screen provided by training instructors. Trainees who had a positive screen were interviewed by medical providers to determine whether further testing was needed.

All laboratory testing (Biofire Respiratory Panel, rapid influenza, and SARS-CoV-2) was conducted on the base. A nasopharyngeal swab was collected for polymerase chain reaction (PCR) testing, and the trainee was isolated in a single-occupancy room and received daily visits from a health care provider or technician to monitor signs and symptoms and determine whether additional care was needed. Isolation rooms were already in place, having been established for previous quarantine of travelers from cruise ships. Symptomatic recruits could return to training at least 7 days after symptom onset and after at least 3 afebrile days.*

## Nonpharmaceutical Interventions

To reduce exposure risk, beginning on March 11, access to the base was limited to essential personnel (Figure). BMT graduation ceremonies, which typically draw family members from around the world, were closed to all visitors. On March 13, training instructors were placed under local area travel restriction to prevent travel-related infection and potential spread to trainees.

**FIGURE:**
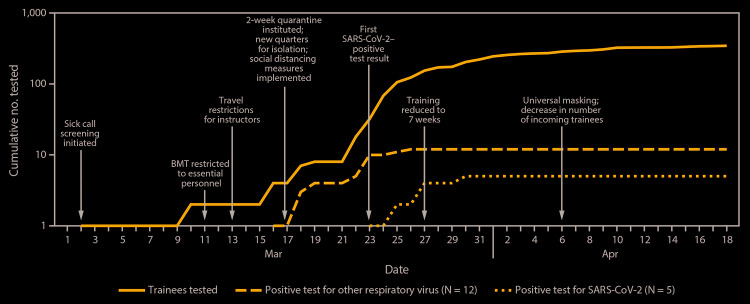
Cumulative number of tested trainees with respiratory symptoms and positive test results for SARS-CoV-2 or other respiratory viruses* and interventions implemented — Joint Base San Antonio-Lackland, Texas, March 1–April 18, 2020 **Abbreviation:** BMT = basic military training. * Rhinovirus or enterovirus (five cases), parainfluenza (three cases), metapneumovirus (two cases), and influenza B virus, (two cases).

Beginning March 17, all new recruits were segregated upon arrival for a 2-week arrival quarantine on an area of the base separated from the main cohort of trainees. In addition, all trainees were instructed to maintain a distance of at least 6 feet between one another to ensure social distancing. After the first positive result in a trainee on March 23, the training schedule was shortened from 8.5 to 7 weeks to maximize efficiency while limiting time of possible exposure. On April 6, universal use of cloth face coverings was introduced. Although BMT had the usual number of incoming trainees through March, BMT stopped taking recruits from areas of the United States with higher community transmission of SARS-CoV-2 in April, reducing the number of incoming trainees by approximately 40%.^†^

## COVID-19 Cases

A total of 10,579 trainees were present at JBSA for BMT during the study period, including 4,073 (39%) who had begun training during March 1–April 18. During that period, 345 (3%) trainees met criteria for testing and further investigation. Among these, 86 (25%) were tested during arrival quarantine, and five (1%) tested PCR-positive for SARS-CoV-2. Testing also identified five cases of rhinovirus or enterovirus, three cases of parainfluenza, two cases of metapneumovirus, and two cases of influenza B. All patients who had positive test results for SARS-CoV-2 or influenza were in arrival quarantine when tested. Public health officials conducted contact tracing for all PCR-confirmed COVID-19 cases.

Patient A arrived at BMT on March 17 and developed symptoms 5 days later, on March 22. The patient was evaluated and found positive with the SARS-CoV-2 PCR test at sick call on March 23 and was immediately isolated. Patients B, C, and D were contacts of patient A during training; they became symptomatic and were evaluated on March 25, March 27, and March 30, respectively. All three had positive test results for SARS-CoV-2. Investigators could not identify the source of infection for patient A; they speculated that he might have been infected during transit because he arrived from a state not reporting community spread of COVID-19.

Another trainee, patient E, arrived at BMT on March 25 and developed symptoms the same day. The patient was evaluated at sick call 2 days later and had a positive test result for SARS-CoV-2. Public health investigations revealed that during the weekend preceding BMT, the patient had visited a large city experiencing community COVID-19 transmission.

All five cases occurred in men. None of the patients required hospitalization or received antimicrobials. Each was placed in isolation until he met the criteria for returning to training. No additional cases were detected during March 1–April 18.

## Discussion

During March 1–April 18, a total of 4,073 incoming trainees joined 6,506 trainees who had already started BMT. Five cases of COVID-19 were diagnosed among incoming recruits, including three cases of transmission within JBSA (cumulative incidence = 47 per 100,000 persons). A combination of administrative controls, increased testing, and quarantine and isolation allowed military training to continue, albeit with 40% reduced numbers, during the first months of the COVID-19 pandemic.

Despite the high risk for transmission in congregate settings (*2*–*4*) such as BMT, as of April 18, the cumulative incidence of symptomatic COVID-19 at the base was lower than the overall rate in the United States (220 per 100,000).^§^ Reports of outbreaks in other congregate settings have been substantially higher. For example, the rates at the base during BMT were significantly lower than the incidence of COVID-19 in homeless shelter residents of 17,000–66,000/100,000 persons over the same time period (*8*).

Despite the communal nature of BMT, which has historically been conducive to outbreaks of respiratory pathogens (*9*), the spread of COVID-19 among trainees in BMT appears to have been low: all cases detected occurred during an initial 14-day arrival quarantine, with no cases identified in the larger training population. Factors contributing to lack of transmission likely included early implementation of mitigation strategies before the first case occurred, mobilization of nonmedical personnel to assist in symptom screening, and flexibility of the military training staff to adjustments in programs and schedules. JBSA had recently accommodated cruise ship passengers during their quarantine, and the infrastructure that was developed to host these passengers was repurposed for BMT quarantine and isolation.

The findings in this report are subject to at least two limitations. First, the interventions were implemented in a highly structured and sufficiently resourced military base. Therefore, the success of these interventions in preventing transmission of SARS-CoV-2 at JBSA might not be transferrable to other settings. Second, cases in asymptomatic or presymptomatic persons cannot be detected by symptom screening, and the prevalence of asymptomatic SARS-CoV-2 infection in this young population with few underlying medical conditions is unknown. However, because no COVID-19 cases were identified during training after quarantine, asymptomatic transmission within this cohort is unlikely. Studies to assess potential asymptomatic spread in military facilities setting are needed.

Transmission of symptomatic COVID-19 was successfully limited at a single military base with adequate resources to screen personnel and the ability to track the movement of all trainees. Despite the presence of 10,579 persons from across the country in communal residence and training, early interventions focusing on NPIs including quarantine, physical distancing, and source control (universal use of cloth face coverings), along with rapid identification and isolation of potential cases, permitted continuation of operations at JBSA during the COVID-19 pandemic. The disciplined and highly structured environment and the population structure (young healthy adults) likely contributed to the success of the implemented interventions. These findings demonstrate the success of widespread implementation of NPIs focused on social distancing, quarantine, and source control in preventing transmission of SARS-CoV-2.

**Summary**

**What is already known about this topic?**

Substantial COVID-19 transmission has been documented in some congregate living settings.

**What is added by this report?**

Nonpharmaceutical interventions (NPI) introduced among 10,579 basic trainees at Joint Base San Antonio-Lackland limited COVID-19 incidence to five cases (47 per 100,000 persons), three of which were in persons who were contacts of the first patient.

**What are the implications for public health practice?**

Despite documented outbreaks of COVID-19 in congregate settings, implementation of NPIs, including screening, testing, administrative measures, quarantine, isolation, and source control, can limit transmission of symptomatic COVID-19 and ensure continuity of critical activities.

## Footnotes

* https://www.cdc.gov/coronavirus/2019-ncov/hcp/return-to-work.html.

† https://www.defense.gov/Explore/News/Article/Article/2145502/precautions-protect-air-force-trainees-from-covid-19/.

§ https://www.cdc.gov/coronavirus/2019-ncov/cases-updates/cases-in-us.html.
